# Whole-Genome Sequence of Cervid atadenovirus A from the Initial Cases of an Adenovirus Hemorrhagic Disease Epizootic of Black-Tailed Deer in Canada

**DOI:** 10.1128/mra.00662-22

**Published:** 2022-09-21

**Authors:** Oliver Lung, Mathew Fisher, Michelle Nebroski, Glenna McGregor, Helen Schwantje, Tomy Joseph

**Affiliations:** a National Centre for Foreign Animal Disease, Canadian Food Inspection Agency, Winnipeg, Manitoba, Canada; b Department of Biological Sciences, University of Manitoba, Winnipeg, Manitoba, Canada; c Animal Health Centre, Ministry of Agriculture and Food, Abbotsford, British Columbia, Canada; d Ministry of Forests, Lands, Natural Resource Operations, and Rural Development, Nanaimo, British Columbia, Canada; Portland State University

## Abstract

A complete 30,616-nucleotide Cervid atadenovirus A genome was determined from the tissues of black-tailed deer that had died in 2020 in British Columbia, Canada. Unique, nonsynonymous single-nucleotide polymorphisms in the E1B, Iva2, and E4.3 coding regions and deletions totaling 74 nucleotides that were not observed in moose and red deer isolates were present.

## ANNOUNCEMENT

**FIG 1:**
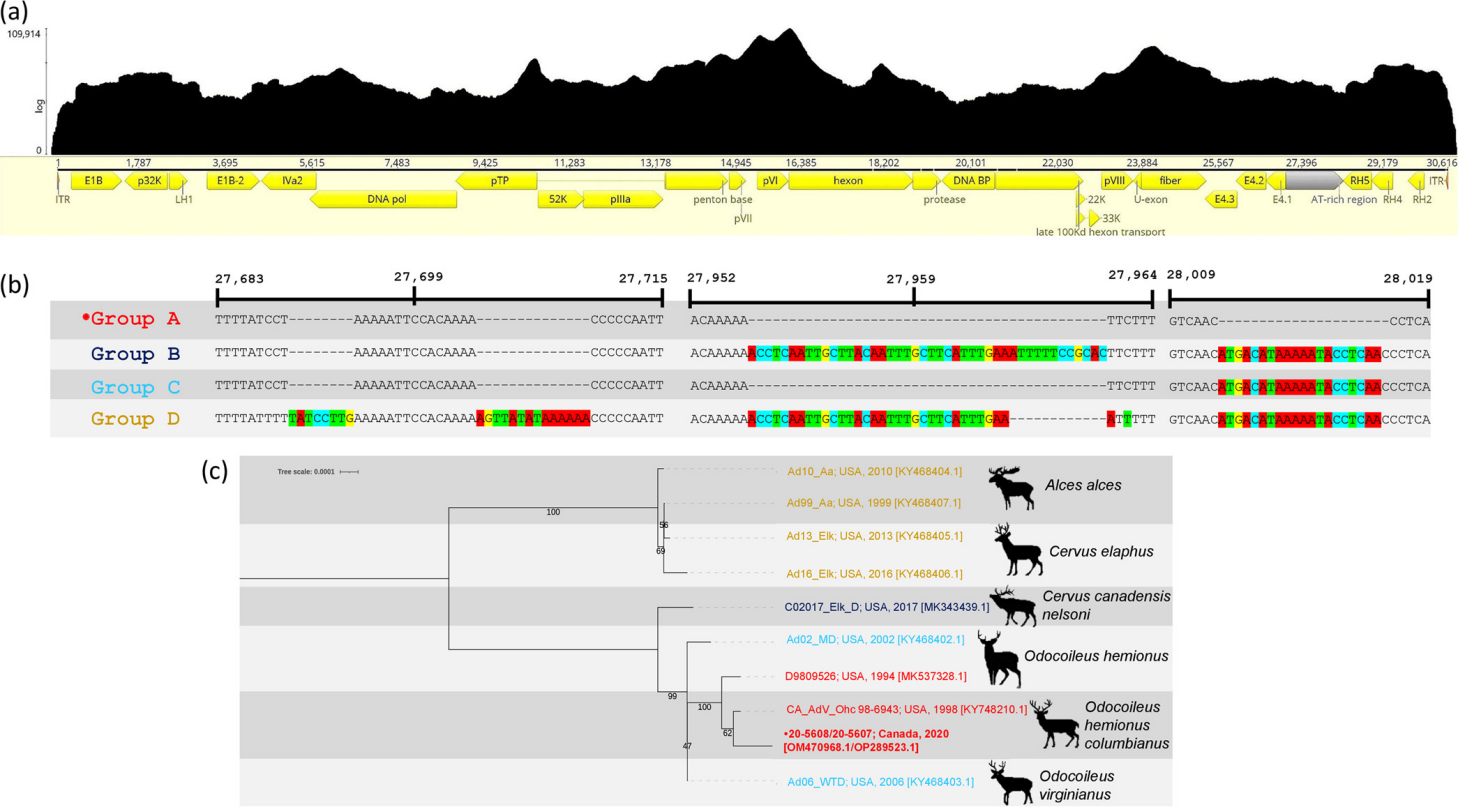
(a) Genome organization of Cervid atadenovirus A, showing coverage depth based on mapping of reads from one sample to the assembled complete genome. (b) Nucleotide alignment of the variable region of the AT-rich noncoding region of 10 Deer atadenovirus A genomes. Genome position numbering, insertions, and SNPs are relative to the Cervid atadenovirus A from this study (20-5608), with insertions and SNPs highlighted. Genomes with identical sequences are grouped together. Group A contains accession numbers OM470968, OP289523, MK537328, and KY748210, group B contains accession number MK343439, group C contains accession numbers KY468402 and KY468403, and group D contains accession numbers KY468407, KY468404, KY468405, and KY468406. (c) Midpoint-rooted maximum likelihood phylogenetic tree of Cervid atadenovirus A (Deer atadenovirus A) whole genomes. The genomes for the tree are the same as the accessions listed in panel B. Alignment was performed using MAFFT with default parameters (9), and the tree was generated using IQ-TREE (12) with the find best model setting with ModelFinder (13) (model HKY-F was chosen) and 1,000 ultrafast bootstraps (14). Isolate names of sequences, as well as the location and year of collection and the GenBank accession numbers, are shown, and the species of the host is indicated on the right. The genome presented here is indicated with a bullet.

Adenovirus hemorrhagic disease (AHD) is an acute, infectious, and usually fatal viral disease of several cervid species. AHD was first described in black-tailed deer in California in 1993, although retrospective analysis of tissues suggested that AHD was present in California in 1981 (1). The disease was previously reported in Canada (2), although no genome sequences are available. Deer deaths were reported on Galiano Island, British Columbia, Canada, in September 2020, with subsequent analyses (histopathology, adenovirus consensus PCR, and amplicon sequencing) confirming AHD (3). Since then, hundreds of black-tailed deer on many Gulf Islands, including Vancouver Island, have died of confirmed AHD (4). The disease was also observed in neighboring Washington state (in the United States) in 2021 (5). The 10 currently available complete genomes for *Deer atadenovirus A* (*Odocoileus adenovirus 1* [OdAdV-1]), the causative agent of AHD, were all obtained from cervids in the United States (Fig. 1c).

Total DNA that had been extracted using the QIAamp DNA minikit (Qiagen) from frozen lung tissue samples from three black-tailed deer that had died of AHD on Galiano Island (two initial samples collected on 11 September 2020 and one subsequent sample collected on 16 September 2020) was submitted for high-throughput sequencing (HTS). Preparation of cDNA and subsequent HTS were performed, followed by viral sequence enrichment with a custom capture-probe set (6), following a previously published method (7). Enriched libraries were quantified, pooled, and sequenced on an Illumina MiSeq sequencer with a v2 flow cell and a 500-cycle kit (Illumina).

Default parameters were used for all bioinformatic analyses. The initial exploratory metagenomic analysis was carried out using a custom Nextflow workflow, nf-villumina v2.0.0 (https://github.com/CFIA-NCFAD/nf-villumina), which automates low-quality read removal, read classification, *de novo* assembly, and BLAST homology searches of assembled contigs, as detailed in a previous publication (8). From these results, *de novo* assembled contigs with homology to cervid atadenoviruses were found in all three samples. The presence of complete 40-bp inverted terminal repeats (ITRs) in two of the assembled contigs (one sample from each submission date) indicated that they represented complete genomes. All three contigs were aligned using MAFFT (9) and were identical in overlapping regions. Reference mapping total reads (6,536,978 reads, 7,576,032 reads, and 9,124,356 reads) to the assembled adenovirus genome resulted in mean coverage depths of 109.4× and 4,038.8× for the earlier samples and 4,590.5× for the later sample.

The 30,616-bp assembled adenovirus genomes had a GC content of 33.2%. Comparison with the most closely related OdAdV-1 reference (GenBank accession number KY748210.1) from BLAST and phylogenetic analyses (Fig. 1) showed 99.96% pairwise nucleotide identity, including 100% identity in the ITR. Geneious v9.1.8 (Biomatters) (10) was used for reference mapping, determination of coverage depth, and comparison to a reference sequence. A single-nucleotide polymorphism (SNP) at position 1,409, causing a potential 12-amino-acid truncation, was observed in the viral replication-critical E1B protein (11). Nonsynonymous substitutions were also found in Iva2 (T5486C) and E4.3 (C25817T) coding regions (Table 1). The variable region of the noncoding AT-rich region contained four deletions totaling 74 nucleotides, compared to isolates from moose and red deer, and appears to cluster with black-tailed deer and mule deer isolates (Fig. 1).

**TABLE 1:** SNPs observed between the Cervid atadenovirus A genome and the most closely related publicly available match, OdAdV-1 (GenBank accession number KY748210.1)

Genome position^*a*^	Gene	Nucleotide(s) in OdAdV-1 (GenBank accession no. KY748210.1)	Nucleotide(s) in Cervid atadenovirus A 20-5608	Effect on coding sequence
267	Noncoding region	A	C	No effect
1,409	E1B	G	T	12-amino-acid truncation
5,486	IVa2	T	C	Nonsynonymous (Lys to Glu)
12,796	pIIIa	G	A	Synonymous
14,961	pVII	Y (C or T)	C	No effect if reference is C, but if reference is T then nonsynonymous (Leu to Phe)
15,134	pVII	T	A	Synonymous
16,238	Hexon	C	T	Synonymous
25,817	E4.3	C	T	Nonsynonymous (Met to Ile)
27,054–27,056	AT-rich region	TAT	– – –^*b*^	No effect
27,968	AT-rich region	C	–	No effect
28,364	RH5	C	T	Synonymous
29,295	RH4	K (G or T)	G	No effect if G in reference, but if reference is T then nonsynonymous (His to Asn)

a Genome positions are relative to OdAdV-1 (GenBank accession number KY748210.1).

b Dashes indicate a deletion in the sequence relative to the reference.

### Data availability.

Nucleotide sequences, including annotations and raw sequencing reads for the two complete genome sequences, were deposited in GenBank and the NCBI Sequence Read Archive (SRA) under BioProject accession number PRJNA803320 with the following GenBank, SRA, and BioSample accession numbers: OM470968, SRX14039924, and SAMN25644053 and OP289523, SRX17188812, and SAMN30469495, respectively. The versions described in this paper are the first versions.
